# Amino Acids and Lipids Associated with Long-Term and Short-Term Red Meat Consumption in the Chinese Population: An Untargeted Metabolomics Study

**DOI:** 10.3390/nu13124567

**Published:** 2021-12-20

**Authors:** Fangxu Guan, Wenwen Du, Jiguo Zhang, Chang Su, Bing Zhang, Kui Deng, Shufa Du, Huijun Wang

**Affiliations:** 1 Key Laboratory of Trace Element Nutrition of National Health Commission of China, National Institute for Nutrition and Health, Chinese Center for Disease Control and Prevention, Beijing 100050, China; guanfx1996@163.com (F.G.); duww@ninh.chinacdc.cn (W.D.); zhangjg@ninh.chinacdc.cn (J.Z.); suchang@ninh.chinacdc.cn (C.S.); zhangbing@chinacdc.cn (B.Z.); 2 Key Laboratory of Growth Regulation and Translational Research of Zhejiang Province, School of Life Sciences, Westlake University, Hangzhou 310024, China; dengkui@westlake.edu.cn; 3 Department of Nutrition and Carolina Population Center, University of North Carolina at Chapel Hill, Chapel Hill, NC 27599, USA; dushufa@unc.edu

**Keywords:** red meat, metabolomics, elastic-net regression, biomarkers

## Abstract

Red meat (RM) consumption is correlated with multiple health outcomes. This study aims to identify potential biomarkers of RM consumption in the Chinese population and evaluate their predictive ability. We selected 500 adults who participated in the 2015 China Health and Nutrition Survey and examined their overall metabolome differences by RM consumption by using elastic-net regression, then evaluate the predictivity of a combination of filtered metabolites; 1108 metabolites were detected. In the long-term RM consumption analysis 12,13-DiHOME, androstenediol (3α, 17α) monosulfate 2, and gamma-Glutamyl-2-aminobutyrate were positively associated, 2-naphthol sulfate and S-methylcysteine were negatively associated with long-term high RM consumption, the combination of metabolites prediction model evaluated by area under the receiver operating characteristic curve (AUC) was 70.4% (95% CI: 59.9–80.9%). In the short-term RM consumption analysis, asparagine, 4-hydroxyproline, and 3-hydroxyisobutyrate were positively associated, behenoyl sphingomyelin (d18:1/22:0) was negatively associated with short-term high RM consumption. Combination prediction model AUC was 75.6% (95% CI: 65.5–85.6%). We identified 10 and 11 serum metabolites that differed according to LT and ST RM consumption which mainly involved branch-chained amino acids, arginine and proline, urea cycle and polyunsaturated fatty acid metabolism. These metabolites may become a mediator of some chronic diseases among high RM consumers and provide new evidence for RM biomarkers.

## 1. Introduction

Red meat (RM) has been an important diet component that provides multiple nutrients with high biological value. It has often been the most popular dish on the dining table. During recent decades consumption of RM has increased all over the world, particularly in developing countries, and China has experienced one of the fastest dietary trends toward higher RM consumption [1]. For example, per capita consumption of pork in China has been increasing by 3% every year. According to US Department of Agriculture statistics, in 2011 average per capita pork consumption in China was 38 kilograms (kg) [2].

RM provides all types of essential amino acids, essential fatty acids, micronutrients like iron and zinc, and various vitamins. However, RM consumption is also positively associated with multiple health outcomes, including cardiovascular diseases [3], cancers [4,5], diabetes [3,6], and all-cause mortality [7,8,9]. Nutritional metabolomics is rapidly evolving to integrate nutrition with complex metabolomics data [10]. As it becomes possible to examine concerns about the association between RM consumption and human metabolites, finding and validating biomarkers of RM intake are important to nutritional epidemiology to complement dietary recalls for measuring RM intake and understanding the mechanisms leading to various health outcomes.

A number of researches on biomarkers of food intake are cross-sectional design. Food frequency questionnaires (FFQs), 24 h food recall, or other dietary assessment tools can identify consumers of specific foods. Comparison of these consumer groups can help identify biomarkers that are reflective of habitual intake provided that these biomarkers have sufficient half-life in the organism or that the foods are regularly consumed. Although some studies have shown the potential of cross-sectional studies, one must be aware of the high correlation between foods consumption, creating a risk of identifying biomarkers that are not specific to the particular food of interest. Even so, cross-sectional data are valuable resources that are currently to be developed and digging for dietary biomarker discovery [11].

Research has reported on several putative biomarkers related to RM intake. Acylcarnitines, O-acetylcarnitines [12], carnitine, 3-dehydrocarnitine, anserine, β-alanine, 4-hydroxyproline, histidine, 13C/12C, 15N/14N, carnosine, creatine, 1-methylhistidine, and 3-methylhistidine [13,14] have been found a higher concentration after RM consumption. However, these compounds and their precursors are also associated with the overall intake of any meat, and they, therefore, are not specific to RM intake [15].

This study aimed to identify possible biomarkers of long-term (LT) and short-term (ST) RM consumption in the Chinese population with untargeted metabolomics technology. We also constructed a prediction model using selected metabolites to test the predictive qualities of these potential markers.

## 2. Materials and Methods

### 2.1. Study Population

The China Health and Nutrition Survey (CHNS) is an ongoing nationwide household-based survey begun in 1989 to compile health, nutritional status, and sociodemographic information in the Chinese population [16]. Using cluster stratified multistage random sampling, the CHNS covers 15 provinces and municipalities. Detailed information on this cohort study can be found elsewhere [17]. In our cross-sectional study, we used data from the 2015 CHNS for 500 adults ages 25 to 68 in two neighboring southern inland provinces, Guizhou and Hunan. All participants provided written informed consent prior to the surveys. The Institutional Review Boards of the University of North Carolina at Chapel Hill (Ethics Approval No. 07-1963) and the National Institute for Nutrition and Health, Chinese Center for Disease Control and Prevention (Ethics Approval No. 201524), approved this study’s standards for the ethical treatment of participants.

### 2.2. Serum Metabolomic Profiling

We asked participants to maintain their daily routines for three days then fast for 8 to 12 h before blood collection. Professional technicians collected fasting blood samples using 3 mL gel separation tubes for coagulation and filled record charts. We immediately refrigerated the samples at −2 to 8 degrees Celsius (°C) (the ratio of sample to dry ice is 1:1). We precisely collected 400 microliters of serum with pipette five times. We put one collection into a tube for an automatic biochemical analyzer and the other four into freezing tubes for a centrifuge. We cryopreserved the serum samples, transported them to laboratories within three hours, and stored them at −80 °C in a freezer until we processed them. The collection, processing, and storage of all blood samples followed the same standardized protocol with strict quality control (QC). The College of American Pathologists Laboratory Accreditation Program and the International Organization for Standardization 15189 Program accredited the laboratories.

We used the automated Micro lab STAR^®^ liquid handling system to prepare the samples. We added several recovery standards prior to the first step in the extraction process for QC. We precipitated proteins with methanol with vigorous shaking for two minutes with a Glen Mills Geno/Grinder 2000) followed by centrifugation. We divided the resulting extract into five samples: two for separate reverse phase ultrahigh-performance liquid chromatography tandem mass spectrometry (RP/UPLC-MS/MS) methods with positive ion mode electrospray ionization (ESI), one for analysis by RP/UPLC-MS/MS with negative ion mode ESI, one for analysis by hydrophilic ultrahigh-performance liquid chromatography tandem mass spectrometry (HILIC/UPLC-MS/MS) with negative ion mode ESI, and one for backup.

We also analyzed a number of types of controls together with the experimental samples for quality assurance: A technical copy generated by a small portion of each sample; process blanks and solvent blanks generated by water samples and extracts, and a carefully chosen hybrid of QC standards without interfering measurement into each sample. Experimental samples were randomly examined on the platform with QC samples evenly distributed among them [18].

The untargeted metabolomics analysis used an ACQUITY UPLC (Waters, Milford, MA, USA) and a Q Exactive high-resolution accurate mass spectrometer (Thermo Fisher Scientific, Waltham, MA, USA). We dried and reconstituted the extract in solvents compatible with each of the four methods. Two aliquots using acidic positive ion conditions were chromatographically optimized for more hydrophilic and hydrophobic compounds, respectively. The extract was gradient eluted from a C18 column (UPLC BEH 2.1 mm × 100 mm, 1.7 µm, Waters, Milford, MA, USA) by different elute solvents. The third aliquot used basic negative ion optimized conditions and a separate dedicated C18 column, which was gradient eluted the basic extracts from the column using methanol, water and 6.5 mm ammonium bicarbonate at pH 8.0. The fourth aliquot used negative ionization following elution from a hydrophilic column (UPLC BEH Amide 2.1 mm × 150 mm, 1.7 µm, Waters, Milford, MA, USA) using a gradient consisting of water and acetonitrile with 10 mm ammonium formate at pH 10.8. The scan range covered 70–1000 mass-to-charge ratio (*m/z*) which was slightly fluctuated between each method.

We extracted, peak identified, and QC processed the raw data. Then identified the compounds by matching their quantity and ion characteristics with purified standard compounds in Metabolon Inc’s library. which maintained authenticated standards containing *m/z*, chromatographic data, MS/MS data, and retention time index (RI). We based the RI of each compound on its elution relationship (assuming a linear fit) with two surrounding standards, which were isotopically labeled metabolites given a fixed RI value [19].

Professional analysts used proprietary software to visualize data, confirm the consistency of peak identification among samples and remove assignment false and background disturbance. For each sample, we checked library matches for each compound and corrected them. We used the area under the curve (AUC) to quantify peaks. Each compound was normalized, and log-transformed to obtain the relative concentration of each metabolite. We scaled the data of each chemical to register the median as 1 and filled the missing value by using the minimum detection limit.

### 2.3. Dietary Assessment and Covariate Profiling

During a five-day household visit, all participants provided sociodemographic and behavioral information, such as age, education, smoking, alcohol consumption, and physical activity. We recorded their LT dietary behaviors with an FFQ that asked them to recall food purchase information and every type of food they had consumed in the past 12 months, including take-out meals and food consumed outside the home. We used the questionnaires to determine the frequency of consumption of 9 categories that included 63 types of food. We investigated ST dietary behaviors with a three-day food diary that required that participants record every meal for the three days before we collected their blood samples. None of the participants reported additional protein supplementation or major illnesses. Professionally trained investigators filled in all the questionnaires according to unified standards during face-to-face interviews.

Using the FFQs and three-day food diaries, we calculated the sum of lean and fat pork, lamb/mutton, beef/veal, and processed meat each participant consumed to determine LT and ST RM consumption. [2] We divided the LT RM consumers into three groups: low consumers (LTLC) who had consumed less than 50 grams per day (g/day), middle consumers (LTMC) who had consumed between 50 and 100 g/day, and high consumers (LTHC) who had consumed more than 100 g/day. We divided the ST RM consumers into three groups: low consumers (STLC) who had consumed less than 76 g/day, middle consumers (STMC) who had consumed between 76 and 136 g/day, and high consumers (STHC) who had consumed more than 136 g/day.

### 2.4. Statistical Analysis

We described the baseline characteristics of our study subjects as median and interquartile ranges for quantitative variables and percentages for categorical variables. We performed a Wilcoxon rank-sum test with false detection rate (FDR) correction on each metabolite to determine the difference between each LT and ST group [20]. We considered a two-sided *p*-value after FDR adjustment (*q* value) less than 0.1 significant.

We split the data set into the training set (70%) and the testing set (30%) and conducted multivariate analyses. Because of the high dimensionality and collinearity feature of our data, we applied an elastic-net penalty (performed with R package “glmnet”, glmnet.stanford.edu/articles/glmnet.html) to reduce the dimension and filter the potential metabolite markers for RM consumers by compressing the coefficient of the unimportant variables to zero. This function fits generalized logistic models via penalized maximum likelihood. The regularization path is computed for the elastic-net penalty at a grid of values (on the log scale) for the regularization parameter lambda. We selected the best tuning parameters for alpha and lambda with 10-fold cross-validation using another R package, “caret”. The “caret” package tests different combinations of alpha and lambda values and specifies the best alpha and lambda values that minimize cross-validation error to produce the final elastic-net model.

We selected both *q* value <0.1 and nonzero variables in the elastic-net model (β ≠ 0) and constructed a forward stepwise logistic regression model on the training set to design the best combination of metabolites for predicting RM consumption levels. We used receiver operating characteristic (ROC) curves to evaluate the accuracy of this model [20]. We performed ROC curve analyses for the designed model in training and testing sets to evaluate the combined model biomarker model by the area under the ROC curve.

After that, we applied an enrichment analysis to find the pathway impact of each potential biomarker and identify the main pathway that correlated with RM consumption using MetaboAnalyst 5.0 (www.metaboanalyst.ca, accessed on 12 November 2021). We performed all statistical analyses with R 4.0.5 software (https://www.rproject.org, accessed on 12 November 2021) and SAS 9.4 software (SAS Institute, Inc., Cary, NC, USA).

## 3. Results

Our study included 500 participants, 204 males and 296 females. In both the LT and the ST analyses the high consumer (HC) groups included larger proportions of males compared to the low consumer (LC) groups. The total energy intakes in the middle consumer (MC) and HC groups were significantly different compared to the LC groups according to the Wilcoxon rank-sum test (*p* < 0.05). Table 1 summarizes the baseline characteristics of the participants.

### 3.1. LT RM Consumption Analysis

After calculating each participant’s LT RM consumption, we categorized 158 people as LTLC (<50 g/day), 178 as LTMC (50–100 g/day), and 164 as LTHC (>100 g/day). Baseline characteristics did not differ significantly between the three groups except for total energy intake per day. The LTHC and LTMC groups had higher energy intakes compared with the LTLC group.

In the univariate analysis, only two metabolites in the LTMC group differed from those of the LTLC group after FDR adjustment. In contrast, 39 metabolites were significantly different between the LTHC and LTLC groups (*q* < 0.1) and are useful for further analysis.

Of the 1108 metabolites we detected, we excluded from our multivariate analysis the unidentified or tentative metabolites and those with more than 80% missing values. We included 792 metabolites in the elastic-net regression model. The best tuning parameters after 10-folds of cross-validation of LTHC versus LTLC elastic-net regression models were α = 0.5500 and λ = 0.0841. After fitting the elastic-net model, 52 metabolites had nonzero coefficients, which means they are an important factor in determining. RM consumption. 10 metabolites met our selection standard (*q* < 0.1 and β ≠ 0), including three lipids, two xenobiotics, three amino acids, and two peptides (Table 2).

**Figure 1:**
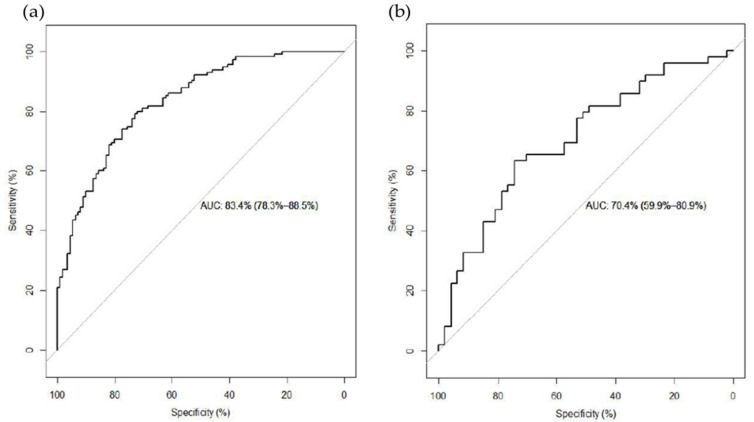
(**a**) Receiver operating characteristic curve of the combination of 10 differential metabolites between long-term red meat low consumers and high consumers in training set. (**b**) Receiver operating characteristic curve of the same metabolites in testing set for prediction.

With these metabolites, we constructed a stepwise logistic regression model adjusted for urban residence, age, gender, and energy intake in the training set as a prediction model. Five metabolites remained in the model: 12,13-DiHOME, androstenediol (3 alpha [α], 17α) monosulfate 2, and gamma-Glutamyl-2-aminobutyrate were positively correlated with HC, while 2-naphthol sulfate and S-methylcysteine were negatively correlated with HC. We evaluated the prediction model by AUC, which was 83.4% (95% CI: 78.3–88.5%) in the training set and 70.4% (95% CI: 59.9–80.9%) in the testing set (Figure 1). When we added four variables to the stepwise logistic model, body mass index (BMI), completed high school, smoking status, and alcohol consumption status. These five metabolites remained significant (*p* < 0.05) in the predictive model and the predictive qualities did not change much. The AUC was 69.9% (95% CI: 59.4–80.4%).

### 3.2. ST RM Consumption Analysis

When we grouped our 500 participants according to their three-day food diaries, 167 were STLC (<76 g/day), 167 were STMC (76–136 g/day), and 166 were STHC (>136 g/day). Our univariate analysis was similar to that of the LT groups. The metabolites in the STMC group showed limited differences when compared with the STLC group. We found that 34 metabolites differed between the STHC and STLC groups.

The elastic-net model selected 173 metabolites with the best tuning parameters α = 0.5500 and λ = 0.0087, and 11 of them met the inclusion criteria (Table 3).

**Figure 2:**
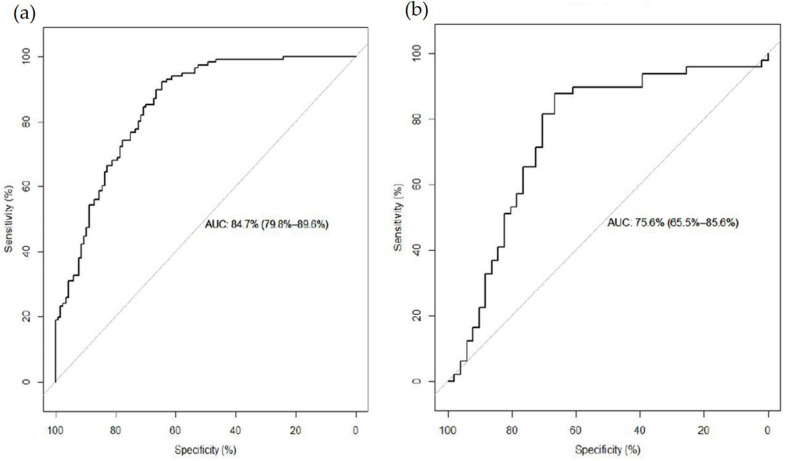
(**a**) Receiver operating characteristic curve of the combination of 11 differential metabolites between short-term red meat low consumers and high consumers in training set. (**b**) Receiver operating characteristic curve of the same metabolites in testing set for prediction.

The stepwise logistic regression model adjusted for urban residence, age, gender, and energy intake showed four significant metabolites. Asparagine, hydroxyproline, and 3-hydroxyisobutyrate were positively associated with ST RM consumption, while behenoyl sphingomyelin (d18:1/22:0) was negatively associated with ST RM consumption. The AUC in the training set was 84.7% (95% CI: 79.8–89.8%) and in the testing set was 75.6% (95% CI: 65.5–85.6%) (Figure 2). When we added BMI, completed high school, smoking status, and alcohol consumption status into the model, these four metabolites remained significant, and the AUC was 74.8% (95% CI: 64.7–85.0%).

### 3.3. Enrichment Analysis

Most of the identified metabolites were registered in the Kyoto Encyclopedia of Genes and Genomes (KEGG) database with identification numbers, and we applied an enrichment analysis using their KEGG identifications. The enrichment analysis shows that the most critical pathways are D-arginine and D-ornithine metabolism in LT RM consumption and branched-chain amino acids (BCAAs), that is, valine, leucine, and isoleucine, biosynthesis in ST RM consumption. In the urea cycle, arginine and proline metabolism plays an important role in both LT and ST RM consumption.

## 4. Discussion

RM consumption is increasing in the Chinese population [21]. In addition, epidemiological evidence indicates that the prevalence of chronic diseases related to high RM consumption is also increasing [2]. Untargeted metabolomic technology can detect a wide range of metabolites. In our study, we used untargeted UPLC-MS/MS technology and a combined metabolites selection strategy to investigate RM consumption in relation to serum metabolites in Chinese adults. We determined our participants’ levels of LT RM consumption with FFQs and their levels of ST RM consumption with three-day food diaries.

We chose an elastic-net logistic regression, which has been widely applied in multi-omics biomarker identification. P. Hernandes-Alonso et al. used elastic-net regression to identify plasma metabolites associated with frequent red wine consumption [22], J.P. Drouin-Chartier et al. used elastic-net regression to identify the plasma metabolites associated with both total and specific dairy consumption [23], and J. Li et al. selected metabolites significantly associated with the Mediterranean diet with an elastic-net regression [24].

We identified 10 metabolites, including three lipids, three amino acids, two xenobiotics, and two peptides, that showed significant differences between LTLC and LTHC and 11 metabolites, including three lipids, six amino acids, and one xenobiotic, that showed significant differences between STLC and STHC. Our results show that differences among serum metabolites were mainly lipids and amino acids, but the candidate biomarkers in LT and ST RM consumption analysis have no similarities. The differential metabolites of our ST analysis seem more convincing, and the ST prediction model has a better AUC.

In the LT RM consumption analysis we found that 12,13-DiHOME was associated with the LTHC group, 12,13 DiHOME is a stimulator of brown adipose tissue activity, which is negatively correlated with BMI and insulin resistance. 12,13-DiHOME can increase brown adipose tissue fuel intake and result in decreased levels of serum triglycerides [25]. KEGG database search revealed that 12,13-DiHOME is involved in linoleic acid metabolism. Linoleic acid is one of the polyunsaturated fatty acids (PUFAs) in animal products. The main PUFAs in RM are the essential fatty acids linoleic acid (n-6) and α-linolenic acid (n-3). The human body can convert α-linoleic acid into long-chain beneficial n-3 fatty acids [26]. Linoleic acid is the precursor of arachidonic acid, which can also modulate inflammatory associated risks factor in chronic diseases. S-methylcysteine sulfoxide was negatively correlated with LTHC. S-methylcysteine sulfoxide, found in Brassica vegetables, is considered a phytoalexin, providing protection against microbial pathogens and herbivores through its degradation by cysteine sulfoxide lyases [27]. The bioconversion of individual phytochemicals in the diet, such as glucosinolates, may confer additional health benefits to the host. Human feces bacteria can reduce the dietary compound S-methylcysteine sulfoxide [28]. 2-oxoarginine is an intermediate of the urea cycle, which was reported as a potential biomarker of arthritis [29].

In the ST RM consumption analysis we observed high concentrations of several amino acids, including asparagine, 4-hydroxyproline, and 3-hydroxyisobutyrate (3-HIB), in the HC group. These amino acids are mainly involved in alanine and aspartate metabolism in the urea cycle, arginine and proline metabolism, and BCAAs biosynthesis. RM contains proteins with various amino acids of high nutritional value for the human body. Our results indicate that ST RM consumption may activate the amino acid metabolism pathway. Asparagine is the metabolic intermediate of alanine, aspartate, and glutamate metabolism; 4-hydroxyproline is a substrate of glycine, pyruvate, and glucose synthesis. Proline is considered a functional amino acid for humans and other mammals; thus, proline is expected to be translated into enhanced efficiency of nutrient utilization and improved health [30]. 3-HIB is a strong marker of insulin resistance in type 2 diabetes and obesity [31]. 3-HIB is a catabolic intermediate of the BCAA valine, secreted from muscle cells, promotes muscle lipid accumulation and insulin resistance in animals, it may cause diabetes as a bioactive signaling metabolite. The high intake of BCAAs is harmful in terms of insulin resistance and Type 2 diabetes [32]. Additionally, lysine is the framework of carnitine synthesis and carnitine can be found in most animal products, especially lambs and beef. Carnitine is an activator not only of fatty acid oxidation but also of carbohydrate metabolism The supplementation of the myocardium with carnitine resulted in an increased tissue carnitine content, a stimulation of pyruvate oxidation, lessening of the severity of ischemic injury and improvement in the recovery of heart function during reperfusion [33].

Other RM biomarker studies have identified several potential biomarkers of RM intake by testing blood or urine samples. In their clinical study, A.B. Ross et al. found that β-alanine and 4-hydroxyproline are potential biomarkers of beef intake among overweight men. Eating beef may also increase concentrations of 2-aminoadipic acid and leucine [34]. In a combined untargeted and targeted metabolomics study, Lu et al. identified a number of amino acids, including hydroxyproline and valine, associated with meat and seafood consumption in a Chinese population [35].

Our study has several limitations. Our participants had no restrictions on their dietary behaviors, so the results of our prediction model may not be satisfying. We categorized participants in the LT RM consumption group based on FFQs that recorded dietary intakes over the past year. That time period may be too long to identify the influence of RM consumption. In addition, recall bias can lower the accuracy of RM consumption reports. Nevertheless, the three-day food diaries may provide relatively accurate ST RM consumption. Additionally, RM consumption is very common among the Chinese population, and consequently, our study participants did not include enough RM non-consumers to compare them with RM consumers.

## 5. Conclusions

In this study, we identified 10 and 11 serum metabolites that differed according to LT and ST RM consumption. We found a higher concentration of amino acids and lipids that act as a mediator in BCAA metabolism, urea cycle, arginine and proline metabolism, and PUFA metabolism. These metabolites may become a mediator of some diseases, especially type 2 diabetes, obesity and cardiovascular disease in high RM consumers. Further research is needed to validate these potential markers as robust biomarkers of RM intake and find the value of these metabolites in investigating the relationship between RM consumption and health outcomes.

## Figures and Tables

**Table 1:** Characteristics of the China Health and Nutrition Survey subjects, median (inter-quartile range) or percentage.

Characteristics		Long-Term			Short-Term	
	Low Consumers (<50 g/day)	Mid Consumers (50–100 g/day)	High Consumers (>100 g/day)	Low Consumers (<76 g/day)	Mid Consumers (76–136 g/day)	High Consumers (>136 g/day)
*N*	158	178	164	167	167	166
Age (years)	53 (47–60)	51 (43–59)	53 (45–60)	53 (46–60)	50 (43–59)	53 (47–59)
Male (%)	29.7	41.6	50.6	31.7	38.9	68.7
Rural (%)	70.9	71.3	58.5	66.5	65.9	51.8
BMI (kg/m^2^) ^a^	23.40 (21.41–26.23)	24.37 (21.70–26.35)	23.83 (21.85–26.03)	23.38 (21.64–26.13)	24.30 (22.23–26.49)	23.75 (21.30–25.87)
Energy intake ^b^ (kilocalories/day)	1625.50 (1348.99–2009.57)	1,974.50 ^c^ (1469.83–2315.49)	2052.50 ^c^ (1619.00–2496.06)	1559.72 (1223.70–2013.81)	1938.71 ^c^ (1507.41–2249.08)	2166.16 ^c^ (1768.74–2599.90)
Completed high school education (%)	19.6	34.3	36.0	25.7	32.3	23.5
Smoker (%)	15.2	24.7	37.2	21.6	23.9	33.7
Alcohol consumer (%)	20.3	20.8	35.4	19.2	21.5	36.8

^a.^ We calculated BMI as weight in kilograms divided by height in meters squared. ^b.^ We calculated total energy intake per day based on three-day food diaries. ^c.^ *p* < 0.05 in the Wilcoxon rank-sum test compared to Low consumers.

**Table 2:** Selected markers of long-term red meat consumption.

Metabolite name		Super Pathway	Sub Pathway	Univariate Analysis		Elastic-Net Model
				*p* ^a^	*q* ^b^	*β* ^c^
12,13-DiHOME ^d^		lipid	fatty acid, dihydroxy	<0.001	<0.001	0.089
2-naphthol sulfate ^e^		xenobiotic	Chemical	<0.001	<0.001	−0.158
androstenediol (3α, 17α) monosulfate 2 ^d^		lipid	androgenic steroid	<0.001	<0.001	0.217
S-methylcysteine sulfoxide ^e^		amino acid	methionine, cysteine, S-adenosylmethionine and taurine metabolism	<0.001	<0.001	−0.130
7alpha-Hydroxy-3-oxo-4-cholestenoate		lipid	Sterol	0.001	0.041	0.008
Perfluorooctane sulfonate		xenobiotic	Chemical	0.001	0.041	0.042
S-methylcysteine		amino acid	methionine, cysteine, S-adenosylmethionine and taurine metabolism	0.001	0.041	−0.014
2-oxoarginine		amino acid	urea cycle, arginine and proline metabolism	0.002	0.065	0.054
gamma-Glutamyl-2-aminobutyrate ^d^		peptide	gamma-glutamyl amino acid	0.003	0.082	0.153
epsilon-(gamma-Glutamyl)-lysine		peptide	gamma-glutamyl amino acid	0.003	0.082	0.126

^a.^ The p value in the Wilcoxon rank-sum test between high consumers and low consumers. ^b.^ The probability after false detective rate adjustment. ^c.^ The coefficient in the elastic-net regression model. ^d.^ Positively correlated (*p* < 0.05) with high consumers in the stepwise logistic regression model. ^e.^ Negatively correlated (*p* < 0.05) with high consumers in the stepwise logistic regression model.

**Table 3:** Selected markers of short-term red meat consumption.

Metabolite name		Superpathway	Sub Pathway	Univariate Analysis		Elastic-Net Model
				*p* ^a^	*q* ^b^	*β* ^c^
3-(4-hydroxyphenyl)lactate		amino acid	tyrosine metabolism	<0.001	<0.001	0.590
asparagine ^d^		amino acid	alanine and aspartate metabolism	<0.001	<0.001	3.235
4-hydroxyproline ^d^		amino acid	urea cycle, arginine and proline metabolism	<0.001	<0.001	0.187
cinnamoylglycine		xenobiotic	food component/plant	0.001	0.053	−0.096
leucine		amino acid	leucine, isoleucine, and valine metabolism	0.001	0.053	0.658
lysine		amino acid	lysine metabolism	0.001	0.053	0.226
tricosanoyl sphingomyelin (d18:1/23:0)		lipid	sphingomyelin	0.001	0.053	−0.329
androstenediol (3α, 17α) monosulfate (3)		lipid	androgenic steroid	0.002	0.073	0.268
S-allylcysteine		xenobiotic	food component/plant	0.002	0.073	0.267
3-hydroxyisobutyrate ^d^		amino acid	leucine, isoleucine, and valine metabolism	0.003	0.094	0.384
behenoyl sphingomyelin (d18:1/22:0) ^e^		lipid	sphingomyelin	0.003	0.094	−0.437

^a.^ The *p* value in the Wilcoxon rank-sum test between high consumers and low consumers. ^b.^ The probability after false detective rate adjustment. ^c.^ The coefficient in the elastic-net regression model. ^d.^ Positively correlated (*p* < 0.05) with high consumers in the stepwise logistic regression model. ^e.^ Negatively correlated (*p* < 0.05) with high consumers in the stepwise logistic regression model.

## Data Availability

The datasets generated and analyzed during the current study are available from the corresponding author (H.W.) on reasonable request.

## References

[1] Lam H.-M., Remais J., Fung M.-C., Xu L., Sun S.S.-M. (2013). Food supply and food safety issues in China. Lancet.

[2] Wolk A. (2017). Potential health hazards of eating red meat. J. Intern. Med..

[3] Micha R., Wallace S.K., Mozaffarian D. (2010). Red and processed meat consumption and risk of incident coronary heart disease, stroke, and diabetes mellitus: A systematic review and meta-analysis. Circulation.

[4] Diallo A., Deschasaux M., Latino-Martel P., Hercberg S., Galan P., Fassier P., Allès B., Guéraud F., Pierre F.H., Touvier M. (2018). Red and processed meat intake and cancer risk: Results from the prospective NutriNet-Santé cohort study. Int. J. Cancer.

[5] Turesky R.J. (2018). Mechanistic Evidence for Red Meat and Processed Meat Intake and Cancer Risk: A Follow-up on the International Agency for Research on Cancer Evaluation of 2015. CHIMIA Int. J. Chem..

[6] Feskens E.J., Sluik D., van Woudenbergh G.J. (2013). Meat consumption, diabetes, and its complications. Curr. Diab. Rep..

[7] Sinha R., Cross A.J., Graubard B.I., Leitzmann M.F., Schatzkin A. (2009). Meat intake and mortality: A prospective study of over half a million people. Arch. Intern. Med..

[8] Larsson S.C., Orsini N. (2014). Red meat and processed meat consumption and all-cause mortality: A meta-analysis. Am. J. Epidemiol..

[9] Zeraatkar D., Han M.A., Guyatt G.H., Vernooij R.W.M., El Dib R., Cheung K., Milio K., Zworth M., Bartoszko J.J., Valli C. (2019). Red and Processed Meat Consumption and Risk for All-Cause Mortality and Cardiometabolic Outcomes: A Systematic Review and Meta-analysis of Cohort Studies. Ann. Intern. Med..

[10] Guasch-Ferre M., Bhupathiraju S.N., Hu F.B. (2018). Use of Metabolomics in Improving Assessment of Dietary Intake. Clin. Chem..

[11] Scalbert A., Brennan L., Manach C., Andres-Lacueva C., Dragsted L.O., Draper J., Rappaport S.M., van der Hooft J.J., Wishart D.S. (2014). The food metabolome: A window over dietary exposure. Am. J. Clin. Nutr..

[12] O’Sullivan A., Gibney M.J., Brennan L. (2011). Dietary intake patterns are reflected in metabolomic profiles: Potential role in dietary assessment studies. Am. J. Clin. Nutr..

[13] Cross A.J., Major J.M., Sinha R. (2011). Urinary biomarkers of meat consumption. Cancer Epidemiol. Biomark. Prev..

[14] Khodorova N.V., Rutledge D.N., Oberli M., Mathiron D., Marcelo P., Benamouzig R., Tome D., Gaudichon C., Pilard S. (2019). Urinary Metabolomics Profiles Associated to Bovine Meat Ingestion in Humans. Mol. Nutr. Food Res..

[15] Cuparencu C., Pratico G., Hemeryck L.Y., Sri Harsha P.S.C., Noerman S., Rombouts C., Xi M., Vanhaecke L., Hanhineva K., Brennan L. (2019). Biomarkers of meat and seafood intake: An extensive literature review. Genes Nutr..

[16] Popkin B.M., Du S., Zhai F., Zhang B. (2010). Cohort Profile: The China Health and Nutrition Survey--monitoring and understanding socio-economic and health change in China, 1989-2011. Int. J. Epidemiol..

[17] Zhai F. (2009). A Follow-Up Study on the Changes of Dietary Structure and Nutritional Status of Chinese Residents.

[18] Wang Y., Sha W., Wang H., Howard A.G., Tsilimigras M.C.B., Zhang J., Su C., Wang Z., Zhang B., Fodor A.A. (2020). Urbanization in China is associated with pronounced perturbation of plasma metabolites. Metabolomics.

[19] Evans A.M., DeHaven C.D., Barrett T., Mitchell M., Milgram E. (2009). Integrated, nontargeted ultrahigh performance liquid chromatography/electrospray ionization tandem mass spectrometry platform for the identification and relative quantification of the small-molecule complement of biological systems. Anal. Chem..

[20] Xia J., Broadhurst D.I., Wilson M., Wishart D.S. (2013). Translational biomarker discovery in clinical metabolomics: An introductory tutorial. Metabolomics.

[21] Huang L., Wang Z., Wang H., Zhao L., Jiang H., Zhang B., Ding G. (2021). Nutrition transition and related health challenges over decades in China. Eur. J. Clin. Nutr..

[22] Hernandez-Alonso P., Papandreou C., Bullo M., Ruiz-Canela M., Dennis C., Deik A., Wang D.D., Guasch-Ferre M., Yu E., Toledo E. (2019). Plasma Metabolites Associated with Frequent Red Wine Consumption: A Metabolomics Approach within the PREDIMED Study. Mol. Nutr. Food Res..

[23] Drouin-Chartier J.P., Hernandez-Alonso P., Guasch-Ferre M., Ruiz-Canela M., Li J., Wittenbecher C., Razquin C., Toledo E., Dennis C., Corella D. (2021). Dairy consumption, plasma metabolites, and risk of type 2 diabetes. Am. J. Clin. Nutr..

[24] Li J., Guasch-Ferre M., Chung W., Ruiz-Canela M., Toledo E., Corella D., Bhupathiraju S.N., Tobias D.K., Tabung F.K., Hu J. (2020). The Mediterranean diet, plasma metabolome, and cardiovascular disease risk. Eur. Heart J..

[25] Lynes M.D., Leiria L.O., Lundh M., Bartelt A., Shamsi F., Huang T.L., Takahashi H., Hirshman M.F., Schlein C., Lee A. (2017). The cold-induced lipokine 12,13-diHOME promotes fatty acid transport into brown adipose tissue. Nat. Med..

[26] Wyness L. (2016). The role of red meat in the diet: Nutrition and health benefits. Proc. Nutr. Soc..

[27] Edmands W.M.B., Gooderham N.J., Holmes E., Mitchell S.C. (2013). S-Methyl-l-cysteine sulphoxide: The Cinderella phytochemical?. Toxicol. Res..

[28] Kellingray L., Le Gall G., Doleman J.F., Narbad A., Mithen R.F. (2021). Effects of in vitro metabolism of a broccoli leachate, glucosinolates and S-methylcysteine sulphoxide on the human faecal microbiome. Eur. J. Nutr..

[29] Shi W., Deng Y., Zhao C., Xiao W., Wang Z., Xiong Z., Zhao L. (2021). Integrative serum metabolomic analysis for preventive effects of Yaobitong capsule in adjuvant-induced rheumatoid arthritis rat based on RP/HILIC-UHPLC-Q-TOF MS. Anal. Biochem..

[30] Wu G., Bazer F.W., Burghardt R.C., Johnson G.A., Kim S.W., Knabe D.A., Li P., Li X., McKnight J.R., Satterfield M.C. (2011). Proline and hydroxyproline metabolism: Implications for animal and human nutrition. Amino Acids.

[31] Nilsen M.S., Jersin R.Å., Ulvik A., Madsen A., McCann A., Svensson P.A., Svensson M.K., Nedrebø B.G., Gudbrandsen O.A., Tell G.S. (2020). 3-Hydroxyisobutyrate, A Strong Marker of Insulin Resistance in Type 2 Diabetes and Obesity That Modulates White and Brown Adipocyte Metabolism. Diabetes.

[32] Jang C., Oh S.F., Wada S., Rowe G.C., Liu L., Chan M.C., Rhee J., Hoshino A., Kim B., Ibrahim A. (2016). A branched-chain amino acid metabolite drives vascular fatty acid transport and causes insulin resistance. Nat. Med..

[33] Pekala J., Patkowska-Sokoła B., Bodkowski R., Jamroz D., Nowakowski P., Lochyński S., Librowski T. (2011). L-carnitine--metabolic functions and meaning in humans life. Curr. Drug Metab..

[34] Ross A.B., Svelander C., Undeland I., Pinto R., Sandberg A.S. (2015). Herring and Beef Meals Lead to Differences in Plasma 2-Aminoadipic Acid, beta-Alanine, 4-Hydroxyproline, Cetoleic Acid, and Docosahexaenoic Acid Concentrations in Overweight Men. J. Nutr..

[35] Lu Y., Zou L., Su J., Tai E.S., Whitton C., Dam R.M.V., Ong C.N. (2017). Meat and Seafood Consumption in Relation to Plasma Metabolic Profiles in a Chinese Population: A Combined Untargeted and Targeted Metabolomics Study. Nutrients.

